# Evaluation of the genotoxicity, cytotoxicity and antimalarial effect of sodium metavanadate *po* in a *Plasmodium yoelii yoelii* infected murine model

**DOI:** 10.1016/j.toxrep.2020.08.006

**Published:** 2020-08-15

**Authors:** Brenda Casarrubias-Tabarez, Norma Rivera-Fernández, Marcela Rojas-Lemus, Nelly López-Valdez, Teresa I. Fortoul

**Affiliations:** a Department of Cellular and Tissular Biology, School of Medicine, UNAM, Mexico City, Mexico; b Posgrado en Ciencias Biologicas, UNAM, Mexico City, Mexico; c Department of Microbiology and Parasitology, School of Medicine, UNAM, Mexico City, Mexico

**Keywords:** Genotoxicity, Antimalarial, Plasmodium yoelii yoelii, Sodium metavanadate, Cytotoxicity

## Abstract

Malaria is a parasitic disease with the highest morbidity and mortality worldwide and antimalarial drug resistance has increased in last two decades. Chloroquine and artemisinin which were usedfor the treatment of malaria are also reported with resistances. Recently, some metallic compounds of ruthenium and iridium have been used as possible therapeutic agents against other parasites such as Leishmania and Trypanosoma cruzi. Organic and inorganic compounds of vanadium such as metavanadate, have been used lately because its therapeutic properties as antineoplastic and hypoglycemic agents. In this study we evaluated the genotoxicity and cytotoxicity of metavanadate per os and its working dose, as a previous step for the future use of metavanadate as anti-parasitic agent in a Plasmodium yoelii yoelii malarial lethal model. Our findings suggest that 10 mg/kg is a safe dose that decreases parasitemia and increases the survival of the Plasmodium yoelii yoelii infected mice with no evidence of genotoxicity, cytotoxicity with the dose selected.

## Highlights

• Oral administration of sodium metavanadate 10 mg/kg decreased parasitemia and increased survival in the *Pyy* mice model.
• Oral administration of 10 mg/kg of sodium metavanadate was neither genotoxic nor cytotoxic in the *Pyy* mice model.
• Sodium metavanadate is proposed as a potential antimalaric agent.

## Graphical abstract

**Figure:**
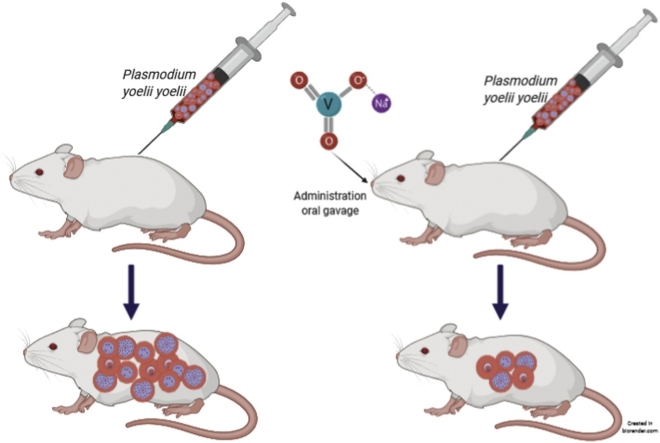

## 1 Introduction

Malaria is the parasitic disease with the highest morbidity and mortality worldwide. In 2018, the World and Health Organization (WHO) reported 219 million cases of clinical malaria and 435,000 deaths. Chloroquine, mefloquine, artemisinin derivatives and some antifolates are still the main antimalarial drugs used around the world [1], nevertheless, pharmacological resistance has increased in the last two decades specially in the endemic areas where the highest rates of morbidity and mortality were observed [2,1]. Chloroquine resistance is observed in Africa and artemisinin combination therapy has been recently reported in Asia. Antiparasitic resistance has led to the design and evaluation of new therapeutic strategies including metals or compounds that bind to metals, like iridium against *Leishmania* [3], ruthenium in *Plasmodium* [4] and vanadium (V) against *Trypanosoma cruzi* [5], *Leishmania* [5,6] and *Plasmodium* [7]. In recent years, organic and inorganic vanadium compounds have gained pharmacological importance due to its therapeutic properties [8,9], for instance, sodium metavanadate (NaVO_3_) (MV) has been proposed as anticarcinogen [[10], [11], [12]] and other vanadium complexes insulin-mimetic agents [13,14]. A detailed review from Šcibior et al., might be consulted for more detailed information about V effects [15]. The use of metals as antiparasitic agents, including MV, appears to be a possible alternative to face drug resistance, therefore, in the present study we evaluated the antimalarial efficacy of MV in murine malaria as well as its cytotoxicity and genotoxicity. The evaluations of new antiparasitic or therapeutic compounds requires toxicological studies in order to avoid genetic damage in the host as these alterations may interrupt the development of the new drug.

## 2 Material and methods

### 2.1 Animals

Animals were obtained from the vivarium of the School of Medicine, UNAM. Male CD1 mice weighing 30 g were used for the experiments. Animals were kept in polyethylene cages where environmental conditions such as temperature, humidity, and filtered air were regulated. The management was performed according to the [16] for the production, care, and use of laboratory animals in accordance to international guidelines. Mice were divided into groups of five mice each. The protocols were approved by the Research and Ethical Committee from the School of Medicine, UNAM (FMED/CI/RGG/094/2016).

### 2.2 Parasites

*P. yoelii yoelii* (*Pyy*) lethal strain was obtained from the London School of Hygiene and Tropical Medicine and maintained by serial passages in CD1 mice.

### 2.3 *In vivo* antimalarial assay antimalarial assay

**Fig. 1:**
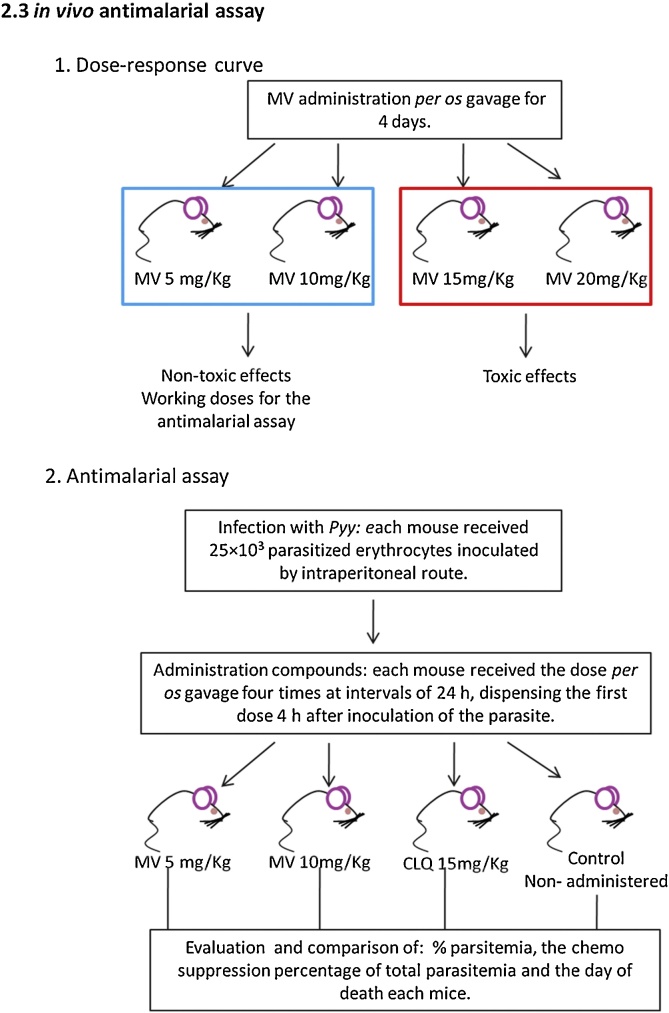
Scheme of the treatment of the animals with sodium metavanadate (MV) and *P. yoelii yoelii* (Pyy). In 1, the method for obtaining the dose-response curve for MV is shown. In each group n = 10. In 2, the Pyy infecting procedure, the MV and chloroquine administration is illustrated. In each group n = 10.

The 4-day suppression test by Peters and Robinson [17] was used to evaluate the effect of MV. Animals were grouped and infected according to Rivera et al. [18], 10 mice for each experimental group was used. MV was administrated *per os* gavage. MV concentrations were obtained based on previously reported MV LD50 (76 mg/kg *per os* and 31 mg/kg intraperitoneal) [19,20]; 5, 10, 15 and 20 mg/kg were evaluated in order to observe toxic side effects and a dose-response curve was made to obtain working doses for the antimalarial assay. Chloroquine was administered orally to the positive control group (15 mg/kg) [21] and *Pyy*-untreated mice remained as the control group. On the fifth day post-infection, a blood smear was made to all infected mice and afterwards, the percent of individual parasitemia was estimated. Blood smears were stained with acridine orange (AO) [22] in distilled water (1 mg/mL) and observed underan epifluorescence microscope (Olympus BH-2, Kansas, USA) at 40 × . Samples were analyzed by duplicated. Images were obtained with Motic Image Advance 3.2 software for Windows. The chemo suppression percentage from total parasitemia was calculated as described by Argotte et al. [23] and compared with parasitemia of non-treated *Pyy* and chloroquine treated groups. Animals from the treated (5 and 10 mg/kg) and control groups were followed up until the end of the experiment (15 days). The day of death was registered for all animals and blood samples were taken in order to observe parasitemia at the time of death. Cured animals were those that survived until the end of the experiment and did not show parasites in the blood smears (Fig. 1).

### 2.4 Fluorochrome-mediated viability assay in leukocytes in peripheral blood

In order to evaluate the percentage of dead and living cells after the MV treatment, a viability cell assay was performed according to Strauss method [24] with some modifications. A concentration of 0.005 g/mL of fluorescein diacetate (FDA) was diluted in acetone, and 0.002 g/mL of ethidium bromide (Et-Br) was diluted in phosphate buffer (PBS) from the stock solutions. To produce a work solution, 15 mL of FDA and 100 mL of Et-Br were added to an amber recipient. This solution was mixed 1:1 with the experimental mice whole blood. The evaluation was made by counting the number of fluorescent leukocytes. Samples were analyzed with anepifluorescence microscope (Olympus BH-2, Kansas, USA) at 40 × . Living cells showed green fluorescence whereas dead ones were identified by the red colorin its nucleus. Cell viability was obtained by counting the green and red cell proportions in 200 cells per field and per mouse. Samples were analyzed in duplicate.

### 2.5 AO Micronucleus test and reticulocytes cytotoxicity

The micronuclei test was done based on the method described by Krishna and Hayashi [25] with some modifications. The micronucleus assay shows chromosome breaking or loss after chemical treatment. Duplicated blood smears were made from the peripheral tail blood of the experimental mice and fixed by methanol immersion for 5 min. Smears were stained with 30 μL of AO (1 mg/mL) in distilled water. Samples were observed in an epifluorescence microscope (Olympus BH-2, Kansas, USA) at 40 × . The proportion of reticulocytes (observed as redcells) was counted per 1000 mature erythrocyte (dark cells) field. Micronucleus (MN) frequency was obtained using 2000 reticulocytes per mouse. The stained MN appeared as an intense green-yellow fluorescence at the same under the epifluorescence microscope. Samples were analyzed by duplicate and colcemid *per os* (10 mL/kg) was used as a positive control.

### 2.6 Comet assay

To evaluate the damage in mice DNA after MV treatment, a pH 13 alkaline version comet assay was performed. This version evaluates the number of single and double strand DNA breaks, alkali-labile sites and single strand DNA breaks associated with late DNA repairment [26,27]. The test was performed according to the method described by Singh et al. [28] with some modifications. Five μL of whole peripheral blood obtained from the experimental animals were mixed with 75 μL of 0.5 % low-melting point agarose and pipetted onto a slide previously covered with 150 μL of regular-melting point agarose and afterwards covered with a coverglass. Slides were again covered with 75 μL 0.5 % low-melting point agarose and immersed in lysis solution for 1 h and then placed on a horizontal chamber electrophoresis. DNA was unwinding for 20 min in an electrophoresis running solution. The electrophoresis was conducted for 20 min at 25 V and 300 mA, after wards the slides were removed and alkaline pH was neutralized with 0.4 M Tris, pH 7.5, Et-Br was then added and covered with a cover glass. DNA migration was analyzed on an Olympus BMX60 microscope with epifluorescence. DNA migration was evaluated in 200 cells per mice. Samples were analyzed by duplicate. Cells were classified into five categories accordingto Rodríguez-Mercado et al. [29]. The measurement of the tail/measurement of the head were used to classify cells as follows: 1, no DNA damage; 2 low DNA damage; 3 medium DNA damage; 4 severe DNA damage and 5 complete DNA damage (death of the cell). These results were expressed as DNA index of migration (MI) according to tail size. Experiments were done by duplicate in independent events.

### 2.7 Statistical analysis

Data were analyzed with Graph Pad Prism (5.0) software. In order to identify differences between groups one-way analysis of variance (ANOVA) and *post hoc* Tukey’s test were used. Data were expressed as mean ± standard error of two independent experiments. Statistical significance was set at p < 0.05. All analyzed data had a normal distribution, and one tailed test was performed. Cell samples were obtained from each group in duplicate and coded in adouble-blind manner.

## 3 Results

### 3.1 Toxicity, cell viability and genotoxicity in non-infected mice

No deaths were reported in the animals that received different doses of MV, nevertheless, with 20 and 15 mg/kg mice showed acute diarrhea and dehydration in a dose-dependent manner, thus 5 and 10 mg/kg were chosen to evaluate antimalarial efficacy. None of these two doses caused cytotoxicity; cell viability remained above 95 % during the 4 days of treatment (Table 1) in all the experimental groups. The 5 mg/kg dose of MV caused slight fluctuations in the migration index (MI) at day three of the treatment. Ten mg/kg did not produce DNA breaks at any time. Positive control group treated with colcemid significantly increased DNA breaks during days 3 and 4 post-treatment (migration rate of 1.05 ± 0.01 and 1.14 ± 0.01 respectively). No significant increase in the frequency of micronuclei was observed with any of the two evaluated doses 5 mg MV (3.73 ± 0.28) and 10 mg (3.13 ± 0.10) in comparison with colcemid positive control group that significantly increased MN frequency. MV evaluated doses did not affect reticulocytes/erythrocytes proportion vs positive colcemid group which decreased proportion of reticulocytes at days three and four post-treatment (Table 1). None of the two doses of MV (5 and 10) showed a cytotoxic effect or genotoxicity, therefore both doses were used to evaluate the antimalarial efficacy.

**Table 1:** Sodium metavanadate cytotoxic and genotoxic evaluation in non-infected mice.

Group	Days	Leukocyte Fluorochrome-mediated viability assay (%)	Comet assay Migration index	Micronucleus frequency/ 2000 reticulocytes	Cytotoxicity Reticulocytes proportion /1000 erythrocytes (%)
Control	0 1 2 3 4	97.08 ± 0.54 96.77 ± 0.61 96.31 ± 0.77 96.77 ± 0.59 97.31 ± 0.62	1.03 ± 0.008 1.01 ± 0.003 1.02 ± 0.006 1.01 ± 0.004 1.02 ± 0.004	2.37 ± 0.31 3.05 ± 0.42 2.80 ± 0.34 2.90 ± 0.28 2.72 ± 0.29	14.67 ± 1.76 14.36 ± 1.10 13.49 ± 0.70 14.06 ± 0.94 13.58 ± 1.10
Sodiummetavanadate 5 mg/ kg	0 1 2 3 4	95.73 ± 1.05 97.00 ± 0.73 96.60 ± 0.65 98.13 ± 0.51 97.93 ± 0.48	1.03 ± 0.013 1.04 ± 0.009 1.04 ± 0.006 1.06 ± 0.009* 1.03 ± 0.006	2.81 ± 0.34 3.53 ± 0.34 3.91 ± 0.36 4.58 ± 0.36 3.85 ± 0.37	14.34 ± 0.63 14.69 ± 0.71 13.11 ± 0.73 14.33 ± 0.64 15.27 ± 0.68
Sodiummetavanadate 10 mg/ kg	0 1 2 3 4	97.25 ± 0.48 95.71 ± 0.65 96.71 ± 0.32 95.12 ± 0.53 95.90 ± 0.57	1.02 ± 0.003 1.02 ± 0.002 1.03 ± 0.005 1.03 ± 0.004 1.03 ± 0.006	2.74 ± 0.30 3.22 ± 0.30 3.13 ± 0.21 3.35 ± 0.30 3.22 ± 0.25	13.54 ± 1.04 14.74 ± 0.60 13.34 ± 0.62 13.97 ± 0.71 13.96 ± 0.44
Positive control Colcemid 10 ml/kg	0 1 2 3 4	96.75 ± 1.03 97.75 ± 1.43 95.00 ± 1.87 97.50 ± 0.50 96.75 ± 0.47	1.04 ± 0.03 1.06 ± 0.04 1.07 ± 0.01 1.05 ± 0.01 1.14 ± 0.01*	2.01 ± 0.53 3.62 ± 0.7 5.33 ± 0.6 5.42 ± 0.73* 5.34 ± 0.63*	15.51 ± 1.24 15.65 ± 1.27 12.2 ± 1.31 11.05 ± 0.89* 11.33 ± 0.98*

Results are the mean ± standard error of two independent studies. *p < 0.05versus control group.

### 3.2 Cytotoxicity and genotoxicity in *Pyy* infected mice treated with MV

No leukocyte cytotoxicity effects were observed with 5 and 10 mg/kg of MV in both treated and non-treated infected mice, cell viability remained above 95 % (Table 2). DNA breaks evaluated in 5 mg treated group significantly increased during 2, 3, and 4 days post-infection vs non-treated infected group. DNA breaks in the 10 mg group, only increased at day 2 post-infection. MI increased in a significant way from day one to day four in the non-treated infected mice. Cytotoxic evaluation mediated by the reticulocytes/erythrocytes proportions significantly decreased in the infected treated MV 5 mg group and in the non-treated infected group (mean of 10.80 ± 0.77 and 11.60 ± 0.64 respectively), whereas no decrease was observed in the 10 mg treated group (Table 2).

**Table 2:** Cytotoxic and genotoxic effect of sodium metavanadate in *Plasmodium yoelli yoelii* infected mice.

Group	Days	Leukocyte Fluorochrome-mediated viability assay (%)	Comet assay Migration index	Micronucleus frequency/ 2000 reticulocytes	Cytotoxicity Reticulocytes proportion /1000 erythrocytes (%)
Control	0 1 2 3 4	97.08 ± 0.54 96.77 ± 0.61 96.31 ± 0.77 96.77 ± 0.59 97.31 ± 0.62	1.03 ± 0.008 1.01 ± 0.003 1.02 ± 0.006 1.01 ± 0.004 1.02 ± 0.004	2.37 ± 0.31 3.05 ± 0.42 2.80 ± 0.34 2.90 ± 0.28 2.72 ± 0.29	14.67 ± 1.76 14.36 ± 1.10 13.49 ± 0.70 14.06 ± 0.94 13.58 ± 1.10
*Pyy*	0 1 2 3 4	97.65 ± 0.35 95.57 ± 0.54 97.14 ± 0.41 95.29 ± 0.59 95.64 ± 0.78	1.04 ± 0.006 1.08 ± 0.014* 1.06 ± 0.007* 1.05 ± 0.015* 1.06 ± 0.015*	3.03 ± 0.21 3.68 ± 0.31 3.87 ± 0.27 2.89 ± 0.23 2.87 ± 0.28	14.82 ± 0.77 14.69 ± 0.87 14.46 ± 0.71 14.67 ± 0.57 11.60 ± 0.64*^a^
*Pyy* + MV 5 mg	0 1 2 3 4	98.00 ± 0.91 95.33 ± 2.02 96.00 ± 2.08 97.67 ± 1.45 97.33 ± 0.66	1.05 ± 0.005 1.05 ± 0.012 1.08 ± 0.014* 1.08 ± 0.021* 1.08 ± 0.020*	3.30 ± 0.32 3.58 ± 0.39 3.37 ± 0.48 2.76 ± 0.44 2.30 ± 0.44	15.96 ± 0.54 16.18 ± 0.97 15.61 ± 0.68 15.35 ± 0.54 10.80 ± 0.77*^a^
*Pyy* + MV 10 mg	0 1 2 3 4	97.75 ± 0.26 96.27 ± 0.82 96.67 ± 0.39 95.93 ± 0.46 95.67 ± 0.64	1.04 ± 0.005 1.05 ± 0.007 1.07 ± 0.015* 1.06 ± 0.011 1.06 ± 0.014	2.91 ± 0.25 2.84 ± 0.25 3.19 ± 0.35 3.24 ± 0.28 2.84 ± 0.28	15.53 ± 0.95 16.24 ± 1.02 14.96 ± 0.83 17.39 ± 1.13 14.09 ± 0.91

Results are the mean ± standard error of two independent studies. *p < 0.05, versus control group. ^a^p < 0.05, versus *Pyy*+ MV 10 mg group.

**Fig. 2:**
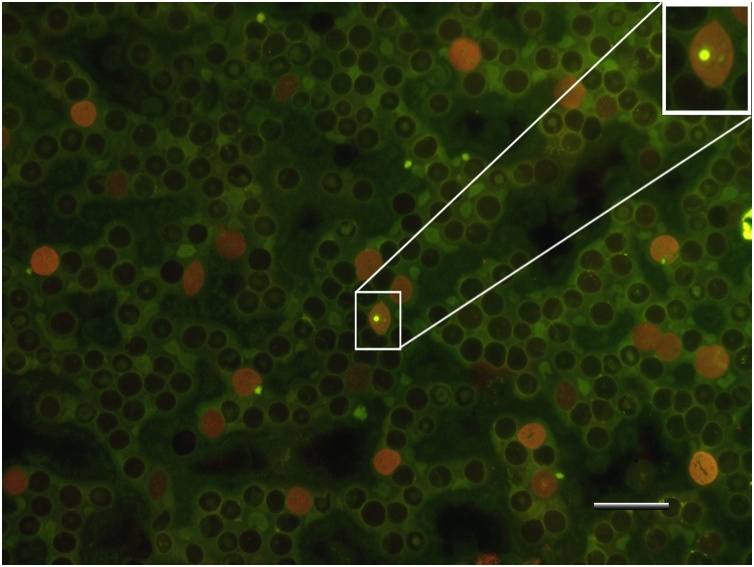
Acridine orange stained peripheral blood smear observed with an epifluorescence microscope. Reticulocytes (red fluorescence), erythrocytes (black cells) and micronucleus (yellow-green fluorescence in square) are observed. Barr 20 μM.

**Fig. 3:**
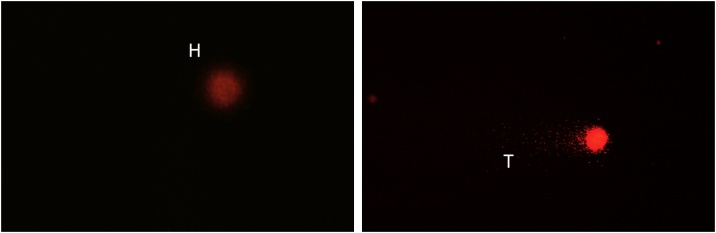
DNA damage categorization in comet assay pH 13 in mice leukocytes. Damage was evaluated as DNA migration from the nucleusbased on the tail/head length. Left image no damage, right image medium damage. H (head), T (tail).

Micronucleus and comet assay images can be observed in Fig. 2, Fig. 3.

### 3.3 Antimalarial effect

**Fig. 4:**
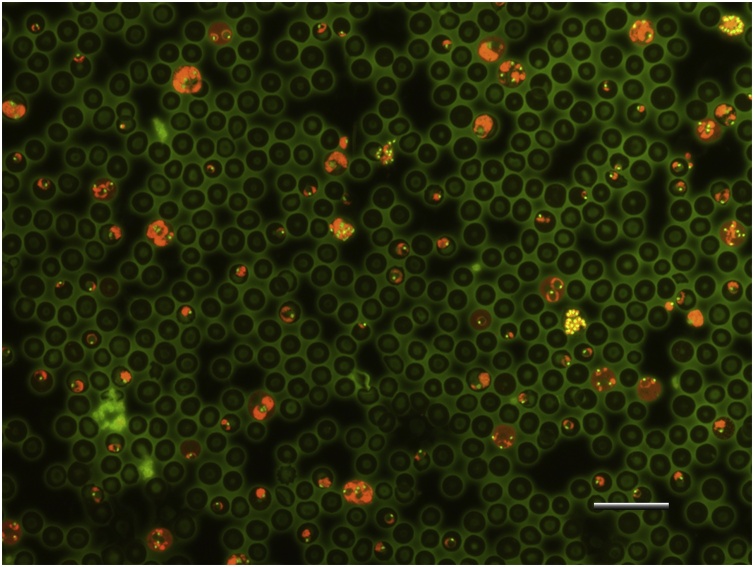
Pyy CD1 infected mice blood smear stained with acridine orange and observed in an epifluorescence microscope. Infected cells are observed: rings (short arrow), trophozoites (long arrow), schizonts (asterisk), merozoites (arrowhead). Parasite was identified based on the staining color: RNA (cytoplasm) red, and DNA (nucleus) bright yellow green. Barr 20 μM.

**Fig. 5:**
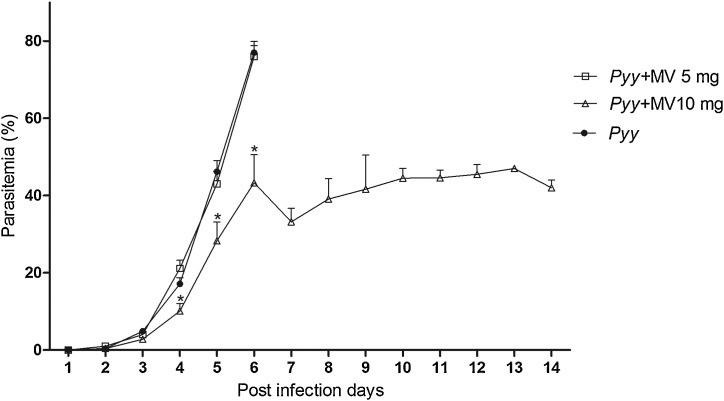
Percentage of parasitemia in mice infected and treated with different doses of sodium metavanadate and infected non treated. Parasitemia of Pyy infected CD1 male mice (●), infected mice treated with MV 5 mg (□) and 10 mg (∆), in this last group a decreased in parasitemia at 4, 5 and 6 (*) days after infection can be observed in comparison with the other groups. ANOVA post-hoc Tukey p < 0.05.

*Pyy* control mice showed parasites in their blood on the fifth day of sampling (46.22 ± 2.79). Animals in this group died at day 6 after infection with a mean parasitemia of 77.00 ± 2.94. Mice from chloroquine control group showed a 100 % chemo suppression of total parasitemia on the fifth day of sampling, so they were considered completely cured. All the animals treated with MV 5 and 10 mg/kg became infected on the fifth day with a mean parasitemia of 43 ± 3.23 and 28.29 ± 4.86 respectively (Table 3). In the mice treated with 10 mg MV, a significant decreased in parasitemia was observed during 4, 5 and 6 days post-infection vs the animals that were treated with5 mg and non-treaded animals. No significant parasitemia reduction was observed in the 5 mg MV group vs the non-treated control group, the mean survival time for this group was 6 days and parasitemia at the day of death was 76 ± 2.79. MV treated mice (10 mg) showed a survival time of 14 days, dying with a mean final parasitemia of 42 ± 2.00 (Fig. 4 and Fig. 5). AO *Pyy* positive blood smears can be observed at Fig. 3.

**Table 3:** In vivo antimalarial efficacy of sodium metavanadate in *Plasmodium yoelii yoelii* infected mice.

Group	Mice number	5th day parasitemia	5th day parasitemia chemo suppression (%)	Parasitemaia at death time (%)	Survival time (days)
*Pyy*	10	46.22 ± 2.79	0	77.00 ± 2.94	6
*Pyy* + MV 5 mg	10	43 ± 3.23	4.43 ± 3.17	76 ± 2.79	6
*Pyy* + MV 10 mg	10	28.29 ± 4.86*	38.79 ± 3.48*	42 ± 2.00*	14
Cloroquine	10	0*	100*	0*	30

Results are the mean ± standard error of two independent studies. *p < 0.05, versus *Pyy*and *Pyy*+ MV 5 mg groups.

## 4 Discussion

Our results revealed that the mice did not show clinical signs neither cytotoxicity effects in peripheral leukocyte viability nor reticulocyte/erythrocyte proportion by MV administration at doses of 5 and 10 mg/kg.

In the antimalarial assay, MV 5 and 10 mg, did not generate leukocyte cytotoxicity in none of the infected groups; At day four of infection, reticulocyte/erythrocyte proportion significantly decreased in *Pyy* and *Pyy*-MV 5 mg groups. No parasitemia decrease was observed in the mice treated with MV5 mg and survival time was six days in comparison with MV 10 mg which showed a partial significant suppression of total parasitemia and survival time was 14 days.

Approximately 435,000 global deaths due to malaria were estimated in 2017 being children under 5 years, immunocompromised patients and pregnant women the most vulnerable groups [30]. Furthermore, antimalarial resistance is one of the main threats for malaria control and therefore the discovery of new antimalarial drugs and drug targets are a fundamental need, as antimalarial vaccines are still in phase III of trials [31]. Some metals have been reported to have an anti-parasitic efficacy [3,5,6]. In our laboratory, CD-1 male mice infected with *Pyy* were exposed to V inhalation, and in preliminary results after a three-day exposure, the exposed mice showed a drastic decrease in parasitemiaand increased survival compared with the infected untreated controls that had 100 % mortality rate [7]. Considering published data regarding the use of metal compounds against protozoa and our previous experience with V in *Pyy* infected mice, in the present study we decided to evaluate MV in the same murine model which can be administrated orally and has a lower absorbance rate in the gastrointestinal tract, feature that is less toxic to the host [8].

In the toxicity assay that was made in order to obtain working doses, mice presented diarrhea with MV of 15 and 20 mg/kg, these results agree with those reported by Llovet et al. [19] and HSE [20] where MV caused intestinal, respiratory and nervous signs with both oral and intraperitoneal LD 50 (31 and 70 mg/kg respectively); in addition, Roberts et al. [32] reported in Sprange Dawley, 18.1 mg/kg/day- and 16.1 mg/kg/day in B6C3F1/ mice, with lower doses compared with those use in this study, clinical manifestations such as: weight loss, ruffled coat, hunched posture, lethargy and abnormal gait. Therefore, 5 and 10 mg were chosen as working doses to continue with the experiments; with these doses, mice did not show clinical signs. MV did not affect peripheral leukocyte viability nor reticulocyte/erythrocyte proportion at the two evaluated working doses (5 and 10 mg). When this proportion is affected, it is because reticulocytes could be dying as a consequence of a toxic factor. This result diverges from the data obtained in anticancer therapies, in which MV has a cytotoxic effect perhaps due to the administered dose (25 μM MV in human pancreatic cancer cell line AsPC-1) [12]; however, Zwolak [43] observed that in non-cancerous cells, cell death was observed in MV doses as high as 600 μM.

No DNA breaks nor MN increase were observed. Some studies showed that sodium orthovanadate and vanadyl sulfate administered orally did not generate genotoxicity [33,34].

In the antimalarial assay, MV 5 and 10 mg, did not generate leukocyte cytotoxicity in none of the infected groups. These results indicate that neither *Pyy* nor MV cause the death of nucleated circulating cells, nevertheless, at day four of infection, reticulocyte/erythrocyte proportion significantly decreased in both groups *Pyy* and *Pyy*-MV 5 mg. It is possible that this decrease is due to the fact that reticulocytes are preferentially parasitized, implying their lysis in the following 24 h after being released from the bone marrow. In addition, the decrease could be related to increased parasitemia and decreased erythropoiesis or suppression reported in malaria infection [35]. No decrease in the proportion of reticulocyte/erythrocyte was observed in MV 10 mg treated mice, this could be due to the fact that in this group there was no increase in parasitemia, therefore there is no destruction of reticulocytes. Short chain DNA (MI breaks increase) were observed in *Pyy* and *Pyy*-MV 5 mg groups but not in *Pyy*-MV 10 mg group. Genotoxic damage could be due to the increased parasitemia in both *Pyy* and *Pyy*-MV 5 mg groups. Increase of reactive oxygen species (ROS) and free radicals generated by the parasite during hemoglobin catalysis [36] as well as the host immune response which also generates ROS during phagocytosis [37,38] might be damaging DNA. Despite the MI increase, there was no change in the micronucleus frequency in both *Pyy* and non-treated *Pyy* groups. This could be related to the generated breaks that were previously repaired by the cell. These results consistently indicate that MV (especially the 10 mg dose) does not cause damage to host DNA, which is a desirable feature in a possible new drug. No parasitemia decrease was observed in mice treated with 5 mg MV, contrasting with all chloroquine treated mice that were cured on the fifth day post-infection. *Pyy* infected mice treated with MV 10 mg, showed a partial significant suppression of total parasitemia (38.79 %) on the fifth day of blood sampling and a survival time of 14 days. Research on the antiparasitic efficacy of vanadium compounds is very recent. No studies were found regarding MV efficacy in malaria, nonetheless, vanadium compounds as oxovanadium have been proven in this and other parasites (Leishmania and Trypanosoma [39] and Entamoeba [40]. Sánchez-Delgado et al. [4] has worked with Ruthenium chloroquine complexes in order to modulate metal activity and prevent resistance. Fricker et al. [41] worked with several metals which exert inhibition of trypanosomatid cysteine proteases. It has been suggested that V has a biological effect due to its ability to interact with biomolecules, therefore ATPases from different protozoa including malaria might be potential targets for V compounds [9,42]. ROS effect could be another action mechanism of V compounds against these protozoans, as they are highly sensitive to oxidative stress [43]. On the other hand, V is found in almost all living organisms and is also present in different food products, and although some toxicity has been reported when used in high doses, in lower doses it seems to be innocuous in comparison to other metals.

In conclusion, 10 mg MV decreases parasitemia and increases the survival time on CD-1 *Pyy* infected male mice in a 4-day test scheme and it is not cytotoxic nor genotoxic to the host. Regular antiprotozoal chemotherapeutic schemes seem to be expanding to include metal compounds and promising results are emerging; thus, these results will allow the continuity of the evaluation of MV as a probable antimalarial agent aiming to identify novel drugs for treatment of parasitic diseases.

## Declaration of Competing Interest

The authors report no declarations of interest.
